# Supplementary material for: Accounting for Defective Viral Genomes in viral consensus genome reconstruction, application to influenza virus
Source: PLoS Comput Biol. 2026 Jul 16;22(7):e1014115. doi: 10.1371/journal.pcbi.1014115 (PMC13395379; doi:10.1371/journal.pcbi.1014115)
Supplement: S1 Appendix — (PDF) [file pcbi.1014115.s001.pdf]

Supplementary Information

Accounting for Defective Viral Genomes in viral consensus genome reconstruction, application to influenza virus

Kévin Da Silva[1,2,3], Nadia Naffakh[4], Marie-Anne Rameix-Welti[1,2,†,*] and Frédéric Lemoine[1,2,3,†,*]

[1]Institut Pasteur, Université Versailles St-Quentin en Yvelines, Paris-Saclay INSERM UMR 1173 (2I), Université Paris Cité, Molecular Mechanisms of Multiplication of Pneumovirus, Assistance Publique des Hôpitaux de Paris, F-75015 Paris, France,

[2]Institut Pasteur, Université Paris Cité, National Reference Center for Respiratory Viruses, F-75015 Paris, France,

[3]Institut Pasteur, Université Paris Cité, Bioinformatics and Biostatistics Hub, F-75015 Paris, France

[4]Institut Pasteur, Université Paris Cité, CNRS UMR3569, RNA Biology and Influenza Virus, F-75015 Paris, France

[†]These authors were co–principal investigators

[*]Corresponding author: frederic.lemoine@pasteur.fr and marie-anne.rameix-welti@pasteur.fr

Table of contents

Supplementary Material and Methods 3

Text A: DIPScan workflow description 3

Text B: Simulated dataset generation and analysis methods 4

Text C: Real dataset analysis methods 5

Supplementary Tables 7

Table A: Description of simulated datasets 7

Table B: Proportion of DelVG falling in the 45-55% range in the real datasets 8

Supplementary Figures 9

Fig A: Illustrative example of the estimation of DelVG + Full length sequences abundance 9

Fig B: Estimated DelVG Proportion (simulated data) 10

Fig C: DelVG Proportion estimation and consensus correction (low coverage simulations) 11

Fig D: Correction of DelVG specific expected mutations across the samples (simulated data) 12

Fig E: Correction of common mutations (simulated data) 13

Fig F: Impact of DIPScan correction on segment phylogenetic trees 14

Fig G: Analysis of potential “breakpoint pairs hotspots” 15

Supplementary Material and Methods

Text A: DIPScan workflow description

Read mapping is performed first with BWA-MEM2, then the unmapped reads and reads with more than 8 clipped positions are mapped with STAR v2.7.11a with the following parameters:

```
STAR --genomeDir ${reference_index} \
--runThreadN ${task.cpus} \
--readFilesIn ${unmapped_fastq} \
--outFileNamePrefix ${prefix} \
--outReadsUnmapped Fastx \
--outSAMattributes Standard \
--outSJfilterOverhangMin 8 8 8 8 \
--outSJfilterCountUniqueMin 3 3 3 3 \
--outSJfilterCountTotalMin 3 3 3 3 \
--outSJfilterDistToOtherSJmin 0 0 0 0 \
--alignSJoverhangMin 8 \
--alignSJstitchMismatchNmax -1 -1 -1 -1 \
--outFilterMismatchNmax 999 \
--alignEndsType EndToEnd \
--outSAMtype BAM SortedByCoordinate
```

Mapped reads from BWA-MEM2 and STAR are eventually merged. In order to differentiate usual short deletions from large defective genome deletions, skipped regions (corresponding to N operation in CIGAR string) shorter than a specific length threshold (default: 150bp) are replaced by deletions (D operation in the CIGAR string) by editing the SAM file obtained from the read mapping.

For the consensus correction, the mapping file (BAM) is filtered to remove spliced reads that do not correspond to selected breakpoints, and SNP calling is performed with iVAR v1.3, with the following parameters:

```
ivar -q 0 -t 0.02
```

Text B: Simulated dataset generation and analysis methods

- Random deletion of a part of a random segment among PB1, PB2, and PA:
```
python random_dips.py -i $fasta -o $output_folder
```
- Addition of random mutations:
```
python random_mutations.py -i $fasta -o $output_folder
```
- Reads simulation:
```
for pc in 10 20 30 40 50 60 70 80 90 100; do nbreads=$((5000000 * $pc /
100)); nextflow run simulator.nf --outdir $output_folder_fulllength --
reference $reference --referencesimu $fulllength --r1 $r1 --r2 $r2 --
adapters $adapters --numreads $nbreads; nextflow run simulator.nf --outdir
$output_folder_dip --reference $reference --referencesimu $dip --r1 $r1 --r2
$r2 --adapters $adapters --numreads $nbreads;  done
```
- Concatenation to create a mix of full-length and defective genomes:
```
for pc in 10 20 30 40 50 60 70 80 90; do
        pc2=$((100 - $pc))
        r1full=$(find $output_folder_fulllength/$pc/ -name "*R1*")
        r2full=$(find $output_folder_fulllength/$pc/ -name "*R2*")
        r1dip=$(find $output_folder_dip/$pc2/ -name "*R1*")
        r2dip=$(find $output_folder_dip/$pc2/ -name "*R2*")
        mkdir -p concat/
        cat $r1full $r1dip > concat/${id}_${pc}-${pc2}_R1.fastq.gz
        cat $r2full $r2dip > concat/${id}_${pc}-${pc2}_R2.fastq.gz
    done
```
- Run DIPScan workflow
```
nextflow run main.nf --samplesheet $samplesheet --consensus $fasta --
output $output_folder
```
- Analyses (simulations/analysis.R)
  - DIP simulated proportions were compared to the estimated proportions computed by DIPScan, either by sample or after averaging the sample results for each DIP simulated proportion.
  - Consensus correction was assessed by aligning the original consensus with the corrected consensus using MAFFT (7.525, --auto parameter) and using a custom script to extract the mutations :
    ```
    python get_mutations_infos.py -i $alignment -o $tsv_output
    ```
  - Mutations were categorized as either common mutations (pre-existing in the sequence used for simulation) or DIP-specific mutations (randomly introduced in the defective genomes). Consensus corrections were classified as correctly changed, wrongly changed, or replaced by ‘N’ (ambiguous).
- Additional downsampled datasets were generated from the initial simulated datasets using fastqutils (github.com/fredericlemoine/fastqutils, v0.1.7) with 10% of the original number of reads (500,000 reads) and 1% (50,000 reads):
```
fastqutils sample \
            --input1  ${sample}_R1.fastq.gz \
            --input2 ${sample}_R2.fastq.gz \
            --number ${number_of_reads} \
            --output1 ${sample}_R1.fastq \
            --output2 ${sample}_R2.fastq \
            --gz
```

Text C: Real dataset analysis methods

- Run DIPScan workflow

```
nextflow run main.nf --samplesheet $samplesheet --consensus $fasta --output $output_folder
```

- Analyses (real_data/analysis.R)
    - We generated two primary dataframes for comparison:
        - A manual curation dataframe with binary columns (0/1) indicating the manually annotated defective status of segment per sample.
        - A DIPScan dataframe from the outputs of DIPScan (breakpoints list), with similar binary columns (0/1) indicating the automatic defective status of each segment per sample. To distinguish critical inconsistencies between manual and DIPScan results, two separate DIPScan dataframes were generated: one including all DIPs, and another including only DIPs representing 50% or more of the genome.
    - Manual and DIPScan data were then merged and compared to produce Fig 7.
    - Breakpoint location analysis (Fig 10) was performed by counting breakpoints by genomic position and segment. Positions along the genome were converted into percentages of segment length. To visualize the distribution of breakpoint counts along each segment, data were first discretized into 0.5% intervals across the full 0–100% coordinate range. For each segment, the number of observations within each interval (bin) was aggregated by summing the counts per bin. The midpoint of each bin was then computed to represent its approximate genomic position. The binned count data were subsequently smoothed using a centered rolling mean with a window size of five bins. Finally, the results were color-coded according to virus subtype.
    - Direct assessment of DIPScan's accuracy in estimating DIP proportions in real viral samples is inherently challenging due to the absence of a known ground truth. Therefore, the output from iVar was used as an indirect proxy for the true DIP proportion. Mutations with frequencies between 10 % and 90 % were extracted, limited to regions outside the defective breakpoints and excluding the 150 bp at both extremities, where coverage is typically lower and frequency estimates can be biased. For each mutation, we attempted to match the mutation frequency with the estimated genome proportion (full-length or DIP). Only mutations that could be unambiguously assigned to a single variant were retained. Results were then averaged by sample and by segment.
    - Direct assessment of DIPScan's accuracy in consensus correction for real viral samples is, once again, inherently challenging due to the absence of a known ground truth. The set of mixtures mutations previously extracted as an indirect proxy for the true DIP proportion was manually curated to retain only mutations that could be unambiguously attributed to DIPs—primarily those carried by reads covering DIP breakpoints. Original and corrected consensus sequences were aligned with MAFFT (7.525, option --auto), but only for segments identified as defective from the breakpoints list. We then extracted the

positions of manually selected mixed mutation sites, and classified the consensus correction as correctly changed, incorrectly changed, correctly unchanged, or replaced by ‘N’ (masked). Unlike the simulation dataset, which featured a controlled, progressive DIP proportion along the x-axis, the selected samples used for consensus correction analysis in real data did not follow a continuous range and were therefore grouped into bins of 10% from 0 to 100% of DIP proportion.

- Phylogenetic trees
  - For each subtype and segment, two phylogenetic trees have been built, one using the corrected sequences, and another one using the corresponding original (uncorrected) sequences.
    - Multiple alignment was realized with MAFFT (7.525, option --auto).
    - Phylogenetic trees were constructed using IQ-Tree v3.1.0 (default parameters).
    - Short branches were collapsed using gotree v0.4.4 with a threshold of 0.1/L with L being the length of the alignment.
  - Phylogenetic trees obtained from corrected and uncorrected sequences were compared by using a tanglegram representation.
    - Trees were formatted using augur (33.1.0):

```
for file in *.align; do
    augur refine \
        --tree ${file}_collapsed.treefile \
        --alignment ${file} \
        --output-tree tmp.tree \
        --output-node-data tmp_refine.node \
        --keep-polytomies
    augur ancestral \
        --tree tmp.tree \
        --alignment ${file} \
        --output-node-data tmp_ancestral.node
    augur export v2 \
        --tree tmp.tree \
        --node-data tmp_refine.node tmp_ancestral.node \
        --output ${file%.align}.json \
        --skip-validation
done
```

    - Resulting json files were uploaded to https://auspice.us

Supplementary Tables

Table A: Description of simulated datasets

Sample	Subtype	Defective segment	DIP breakpoints	#DIP mutations
202503268	A/H3N2	PA	1929-2124	8
202503272	A/H1N1	PA	430-1351	9
202503277	A/H3N2	PB2	415-777	2
202503279	A/H3N2	PB2	893-1583	3
202503284	A/H1N1	PA	1762-2026	10
202503290	A/H1N1	PB2	134-1508	2
202503291	A/H3N2	PA	397-2100	9
202503324	B/VIC	PB1	328-994	5
202503341	A/H1N1	PB2	677-1572	1
202503439	B/VIC	PB1	1913-2269	6
202503448	B/VIC	PB2	1298-2135	3

Table B: Proportion of DelVG falling in the 45-55% range in the real datasets

Subtype	Segment	Prop DelVG<br>in 45-55 range	Number of DelVG
B/Vic	HA	0,00	1
B/Vic	NP	0,00	1
B/Vic	PA	0,21	24
B/Vic	PB1	0,11	113
B/Vic	PB2	0,09	77
A/HINI	HA	0,00	1
A/HIN1	NP	0,00	1
A/H1N1	PA	0,06	66
A/HIN1	PB1	0,09	55
A/HIN1	PB2	0,14	43
A/H3N2	NA	0,25	4
A/H3N2	NS	0,00	2
A/H3N2	PA	0,11	81
A/H3N2	PB1	0,06	82
A/H3N2	PB2	0,05	76

Supplementary Figures

Fig A: Illustrative example of the estimation of DelVG + Full length sequences abundance

a. Available metrics

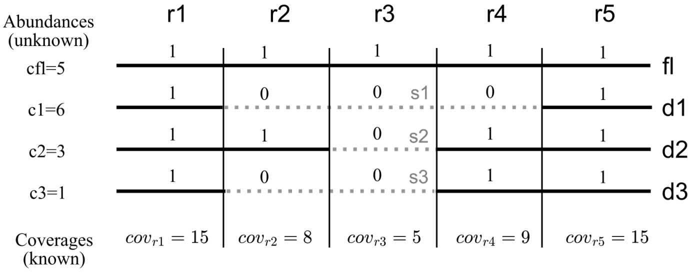

split-reads ratios (known)

$$p_1 = \frac{s_1}{s_1 + s_2 + s_3} = 0.6 = \frac{c_1}{c_1 + c_2 + c_3}$$

$$p_2 = \frac{s_2}{s_1 + s_2 + s_3} = 0.3 = \frac{c_2}{c_1 + c_2 + c_3}$$

$$p_3 = \frac{s_3}{s_1 + s_2 + s_3} = 0.1 = \frac{c_3}{c_1 + c_2 + c_3}$$

b. System of linear equations

$$\Leftrightarrow \begin{bmatrix} 1 & 1 & 1 & 1 \\ 1 & 0 & 1 & 0 \\ 1 & 0 & 0 & 0 \\ 1 & 0 & 1 & 1 \\ 1 & 1 & 1 & 1 \\ 0 & 1-0.6 & -0.6 & -0.6 \\ 0 & -0.3 & 1-0.3 & -0.3 \\ 0 & -0.1 & -0.1 & 1-0.1 \end{bmatrix} \cdot \begin{bmatrix} c_{fl} \\ c_1 \\ c_2 \\ c_3 \end{bmatrix} = \begin{bmatrix} 15 \\ 8 \\ 5 \\ 9 \\ 15 \\ 0 \\ 0 \\ 0 \end{bmatrix}$$

Illustrative example with 3 DIP sequences. **a.** We want to estimate the abundances cfl, c1, c2, and c3, using: i) the coverages covr1,...,covr5 of the 5 regions r1,...,r5, defined by the 3 breakpoints s1, s2, and s3 (corresponding to 4 distinct positions), and ii) the ratios p1, p2, p3 of the number of split reads on s1, s2, s3. The constraint is that these ratios should be close to the ratios of the abundances of the corresponding DIP sequences c1, c2, and c3. **b.** These constraints are formulated as a set of linear equations for which an approximate solution can be found using Non-Negative Least Squares.

Fig B: Estimated DelVG Proportion (simulated data)

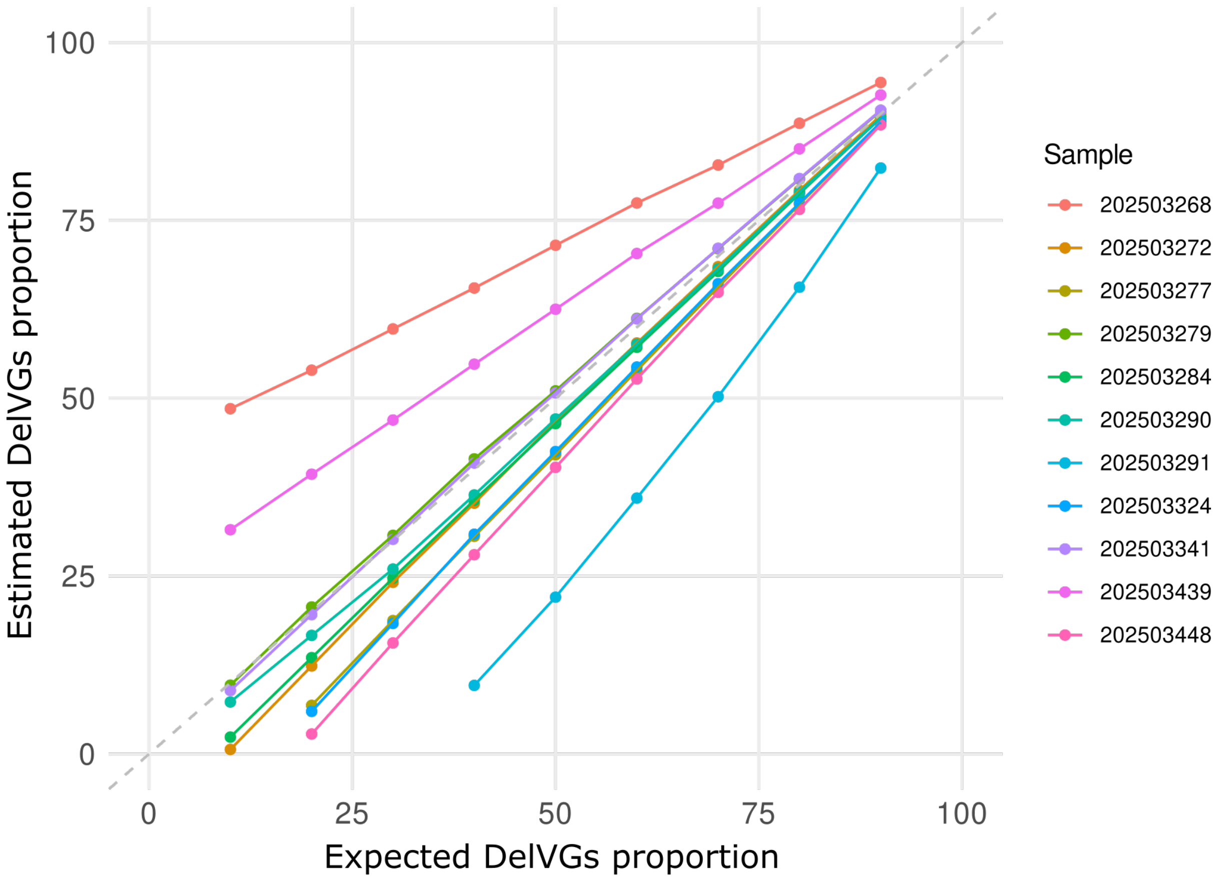

Estimated DelVG ratio at each known (theoretical) DelVG ratio, for the 11 samples individually.

Fig C: DelVG Proportion estimation and consensus correction (low coverage simulations)

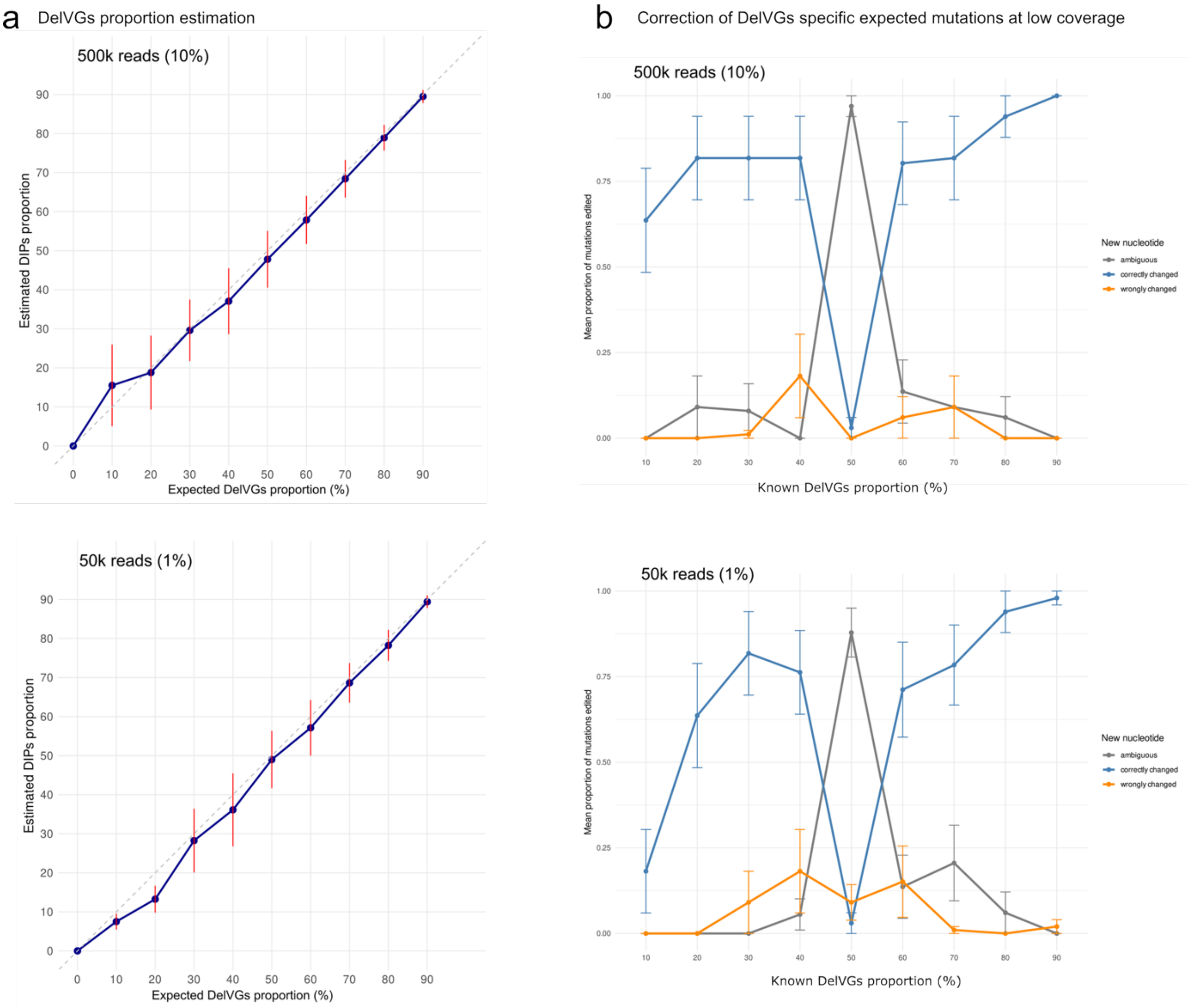

a. Accuracy of DelVG proportion estimation on low coverage simulated datasets. Top: 10% coverage relative to the original simulated samples, bottom: 1% coverage relative to the original sample. The mean distribution and confidence intervals of DelVG ratios estimated by DIPScan (y-axis) are shown for the 11 samples together across each simulated input proportion (x-axis). **b.** Correction performance for DelVG-specific mutations on low coverage datasets. Top: 10% coverage relative to the original samples, bottom: 1% coverage relative to the original simulated samples. The proportion of corrected DelVG-specific mutations (y-axis) as a function of the simulated input DelVG portion (x-axis) is represented for the three following outcomes: replacement with an ambiguous base (gray curve), correct reversion to (or retention of) the full-length genome nucleotide (blue curve), or erroneous retention of the DelVG-specific mutation (yellow curve). As expected, ambiguities peak around a 50% DelVG proportion, while correct retention or reversion peaks at both low DelVG proportion (where the full-length genome is dominant) and high DelVG proportion (where the DelVG is dominant, triggering a reversion to the full-length base).

Fig D: Correction of DelVG specific expected mutations across the samples (simulated data)

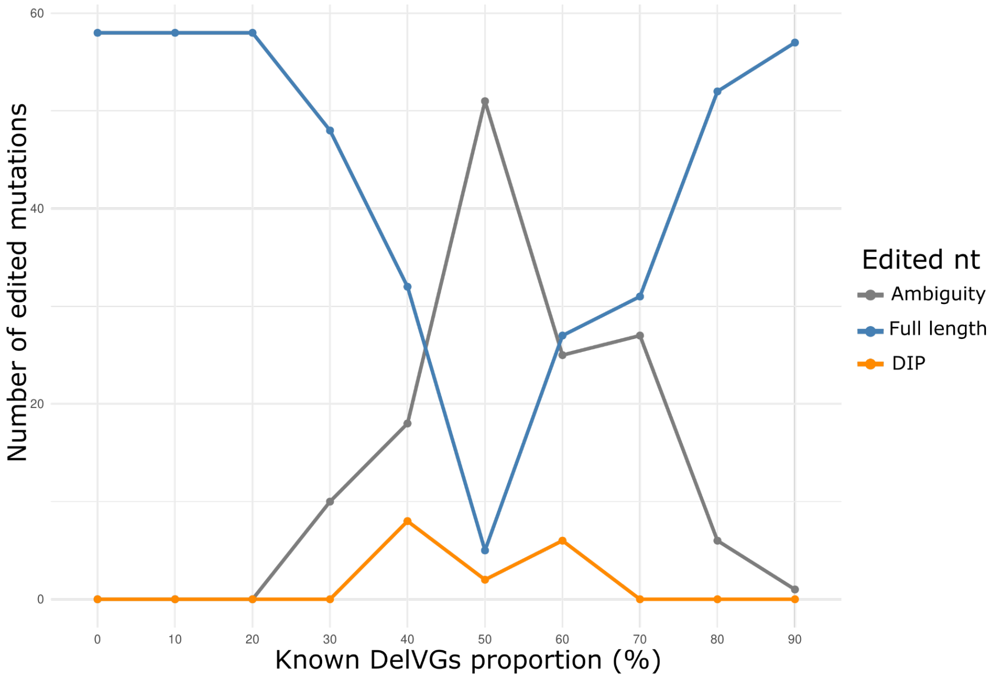

Correction performance for DelVG-specific mutations. The number of corrected DelVG-specific mutations (y-axis) as a function of the simulated input DelVG portion (x-axis) is represented for the three following outcomes: replacement with an ambiguous base (gray curve), correct reversion to (or retention of) the full-length genome nucleotide (blue curve), or erroneous retention of the DelVG-specific mutation (yellow curve). As expected, ambiguities peak around a 50% DelVG proportion, while correct retention or reversion peaks at both low DelVG proportion (where the full-length genome is dominant) and high DelVG proportion (where the DelVG is dominant, triggering a reversion to the full-length base).

Fig E: Correction of common mutations (simulated data)

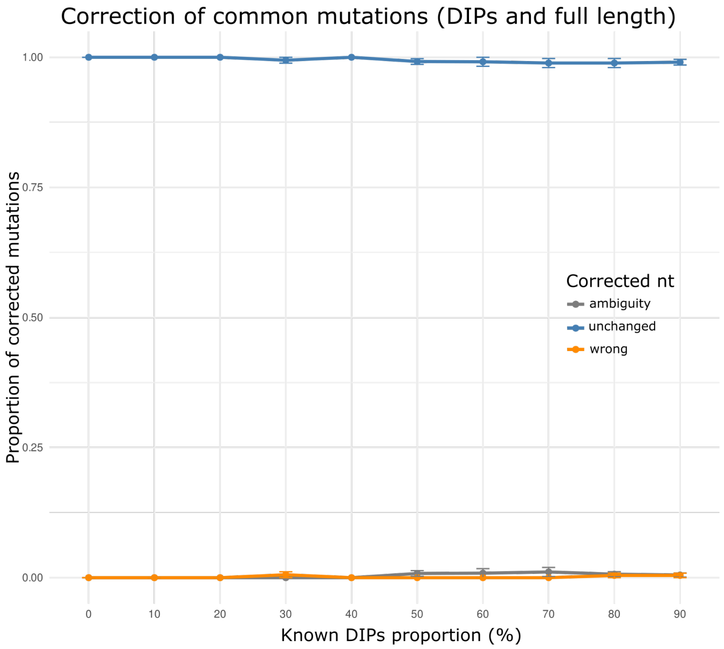

Assessment of the correction of mutations, compared to the reference genome, that are common to the DelVG and the full length sequence. The grey curve corresponds to the cases where the mutation is replaced by an ambiguity, the blue curve corresponds to the unchanged mutations, and the orange curve corresponds to the cases where the wrong nucleotide is incorporated. This shows that cases of wrongly changed positions for common mutations are very rare.

Fig F: Impact of DIPScan correction on segment phylogenetic trees

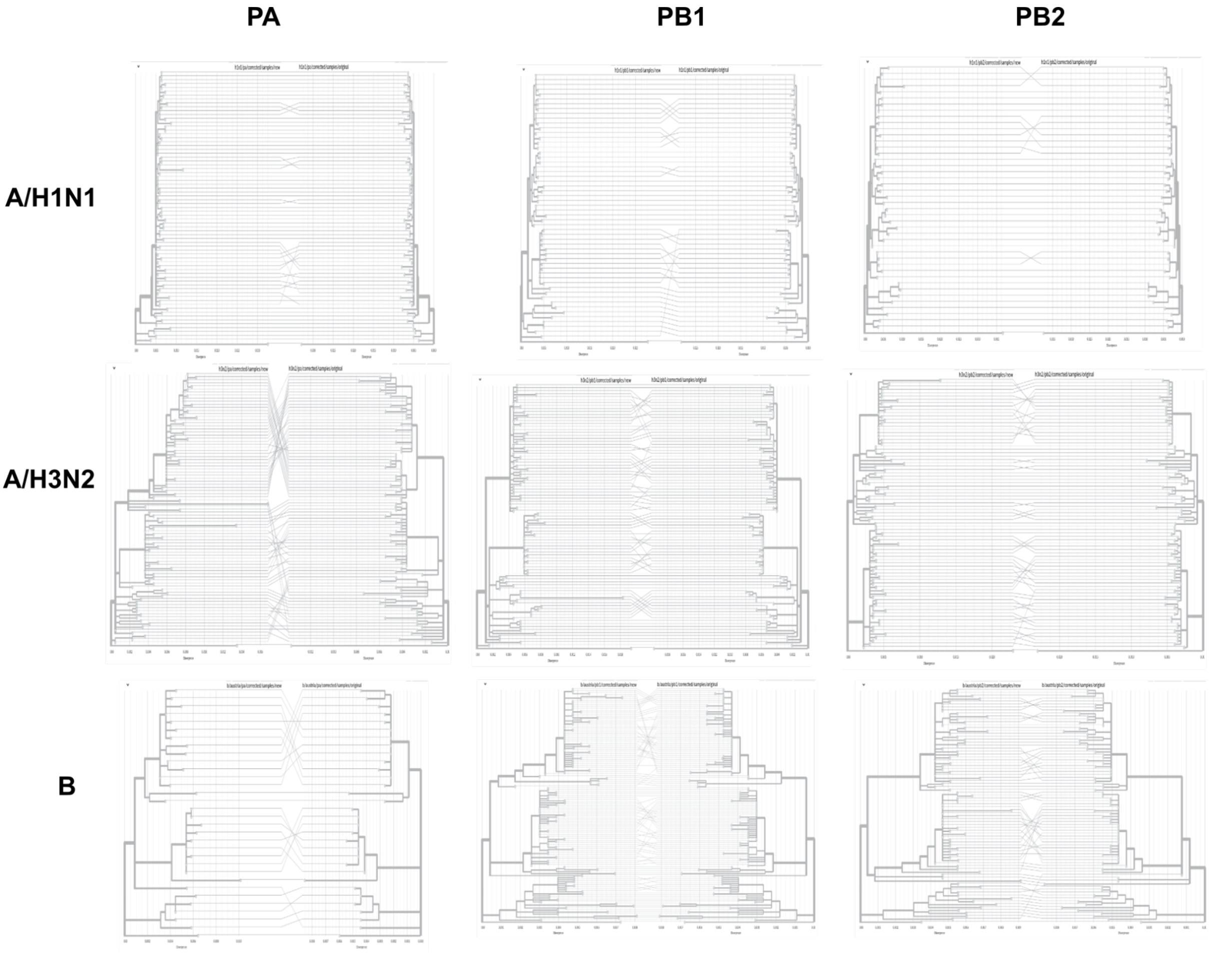

Phylogenetic trees were inferred from all sequences marked as needing correction in the true dataset, before and after DIPScan correction. The trees of the three viruses are shown in rows (A/H1N1, A/H3N2, and B) and the three segments in columns (PA, PB1, and PB2). Trees are represented in pairs, with: before (left) and after (right) correction, as “tanglegrams”, using Auspice.

Fig G: Analysis of potential “breakpoint pairs hotspots”

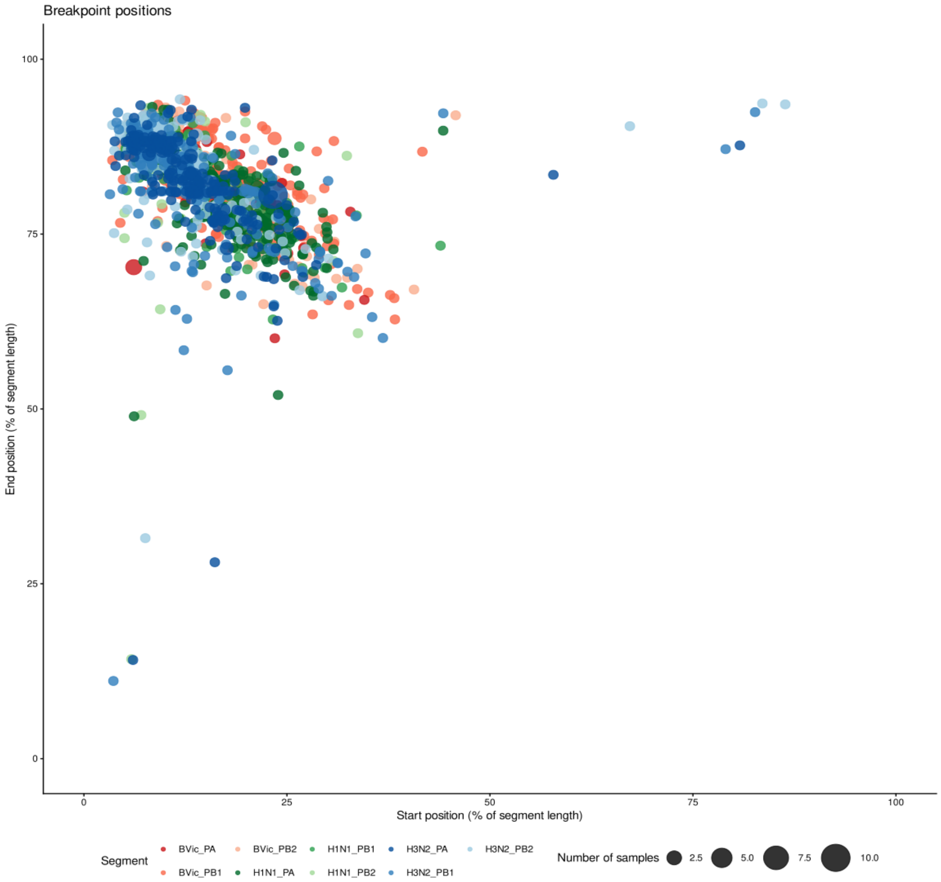

Breakpoint start and end positions (expressed as percentages of segment length) for all 551 samples are plotted on the x- and y-axes, respectively. Points are color-coded by influenza subtype: B/Victoria (red), A/H1N1pdm (green), and A/H3N2 (blue), with light-to-dark shading distinguishing the three predominantly affected segments (PB2, PB1, and PA). The plot reveals distinct breakpoint-pair hotspots across all subtypes and segments, evidenced by a strong linear correlation with points clustering along the diagonal.
